# Supplementary material for: Spray-induced gene silencing for disease control is dependent on the efficiency of pathogen RNA uptake
Source: Plant Biotechnol J. Author manuscript; Available in PMC 2021 Sep 10. (PMC8428832; doi:10.1111/pbi.13589)
Supplement: Supplemental Information — Figure S1 Colony morphology of different types of fungi. Figure S2 Mycelium morphology of different types of fungi. Figure S3 Multiple fungal dsRNA uptake efficiencies were observed over a time course. Figure S4 Accumulation of fluorescein signal intensities over a time course in different fungal cells. Figure S5 Fluorescein-labeled YFP-dsRNA was observed in S. sclerotiorum protoplasts after MNase treatment. Figure S6 Accumulation of fluorescein signal intensities in different fungal cells. Figure S7SsVPS51, SsDCTN1, and SsSAC1 knockout mutants displayed significantly reduced virulence compared to the wild-type strain. Figure S8 Topical application of pathogen gene-targeting dsRNAs had no effect on C. gloeosporioides infection and disease. Figure S9 Accumulation of fluorescein signal intensities in different P. infestans cells. Figure S10YFP-, PiHMP1-, PiPGB1-, PiDCL1-, and Pi-DCTN1+SAC1-dsRNAs did not inhibit P. infestans infection on potato leaves. Table S1 Primers used in the study. Table S2 The sequences of dsRNAs in the study. [file NIHMS1726102-supplement-Supplemental_Information.pdf]

Supplemental Information for

Spray-induced gene silencing for disease control is dependent on the efficiency of pathogen RNA uptake

Lulu Qiao[1,2,3], Chi Lan[1,2], Luca Capriotti[3,4], Audrey Ah-Fong[3], Jonatan Nino Sanchez[3], Rachael Hamby[3], Jens Heller[5,6], Hongwei Zhao[1,2], N. Louise Glass[5,6], Howard S. Judelson[3], Bruno Mezzetti[3,4], Dongdong Niu[1,2,3]*, Hailing Jin[3]*

[1]College of Plant Protection, Nanjing Agricultural University, Nanjing 210095, China

[2]Key Laboratory of Integrated Management of Crop Diseases and Pests (Ministry of Education), Nanjing 210095, China

[3]Department of Microbiology & Plant Pathology, Center for Plant Cell Biology, Institute for Integrative Genome Biology, University of California, Riverside, California 92521, USA

[4]Department of Agricultural, Food and Environmental Sciences, Marche Polytechnic University, 60131 Ancona, Italy

[5]Department of Plant and Microbial Biology, University of California, Berkeley, California 94720, USA

[6]Environmental Genomics and Systems Biology Division, The Lawrence Berkeley National Laboratory, Berkeley, California 94720, USA

*Correspondence:

Dongdong Niu (*ddniu@njau.edu.cn*)

Hailing Jin (*hailingj@ucr.edu*)

This file includes:

Figures S1-S10 and Table S1-S2

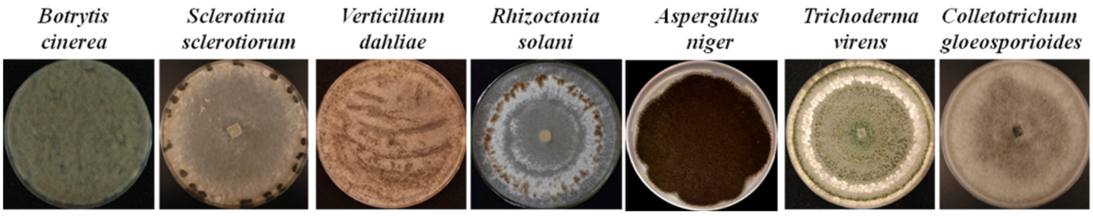

Figure S1. Colony morphology of different types of fungi.

Colony morphology pictures of *B. cinerea*, *S. sclerotiorum*, *V. dahliae*, *R. solani*, *A. niger*, *T. virens*, and *C. gloeosporioides*.

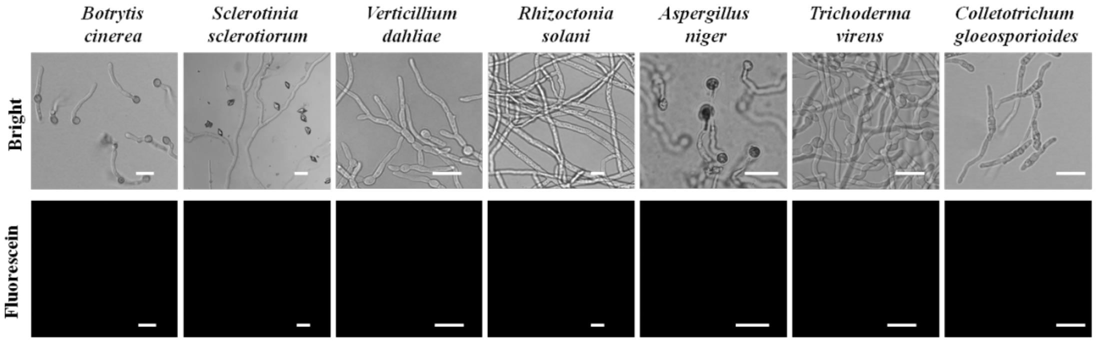

Figure S2. Mycelium morphology of different types of fungi.

Mycelium morphology pictures of *B. cinerea*, *S. sclerotiorum*, *V. dahliae*, *R. solani*, *A. niger*, *T. virens*, and *C. gloeosporioides* were taken by confocal, all fungal cells show no fluorescent signals. Scale bars = 20 µm.

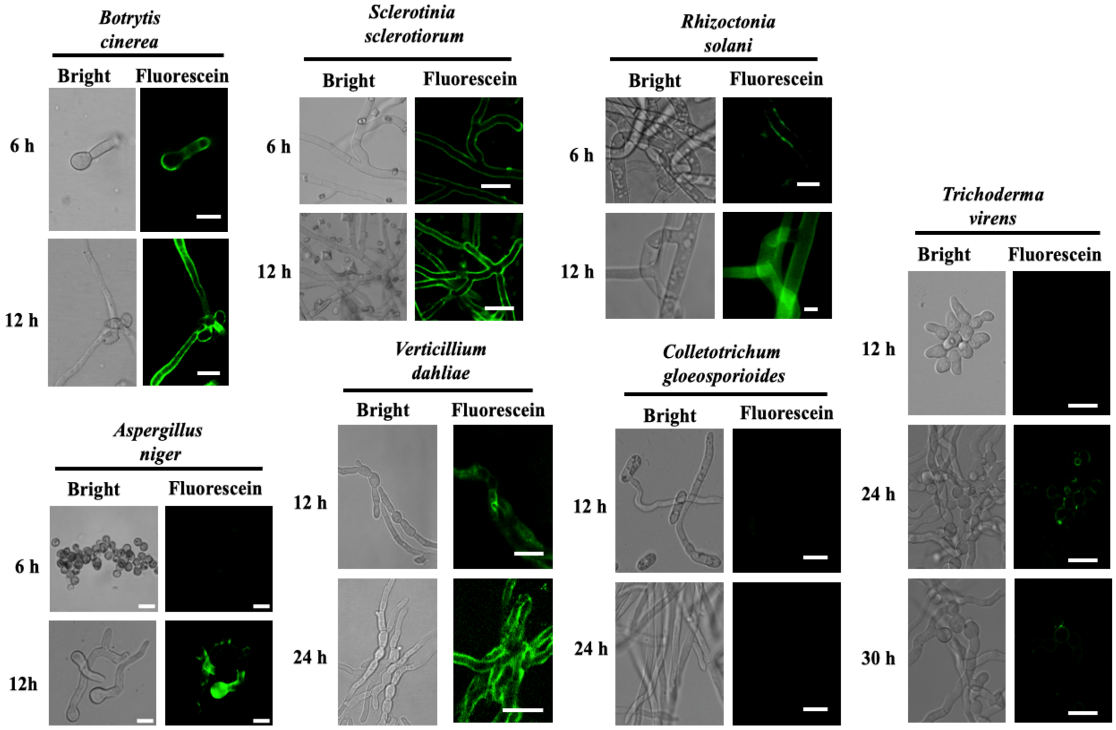

Figure S3. Multiple fungal dsRNA uptake efficiencies were observed over a time course.

Fluorescein-labeled *YFP*-dsRNA was added to *S. sclerotiorum*, *R. solani*, *A. niger*, *C. gloeosporioides*, *V. dahliae*, *B. cinerea*, and *T. virens* hyphae, as well as spores, and the fluorescence signals were observed over time courses uses a Leica confocal microscope. Micrococcal nuclease (MNase) treatment was performed 30 min before image acquisition. Scale bars = 20 µm.

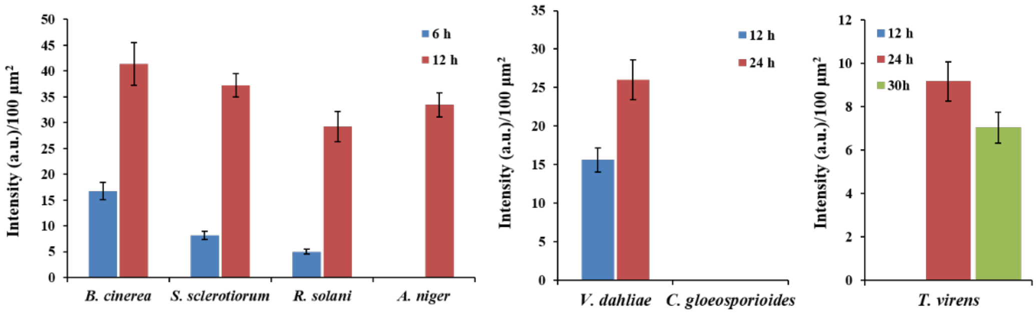

Figure S4. Accumulation of fluorescein signal intensities over a time course in different fungal cells.

Fluorescein-labeled *YFP*-dsRNA was added to *B. cinerea*, *S. sclerotiorum*, *R. solani*, *A. niger*, *V. dahliae*, *C. gloeosporioides*, and *T. virens* hyphae, as well as spores, and the fluorescence signals were observed over time courses uses a Leica confocal microscope. The fluorescein signal intensities were quantified using ImageJ software, which could compare the fluorescence intensity of the same area between different cells over a time course. Similar results are obtained from three biological repeats.

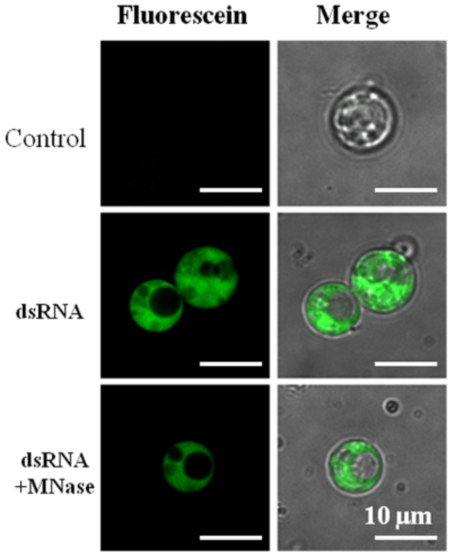

Figure S5. Fluorescein-labeled *YFP*-dsRNA was observed in *S. sclerotiorum* protoplasts after MNase treatment.

Fluorescein-labeled *YFP*-dsRNA was applied to germinated *S. sclerotiorum* mycelium and the protoplasts were isolated after culturing for 48 h. The fluorescent signals were detected within fungal protoplasts and MNase enzyme treatment did not reduce the fluorescent signal intensities. Similar results were obtained over three biological replicates. Scale bars = 10 μm.

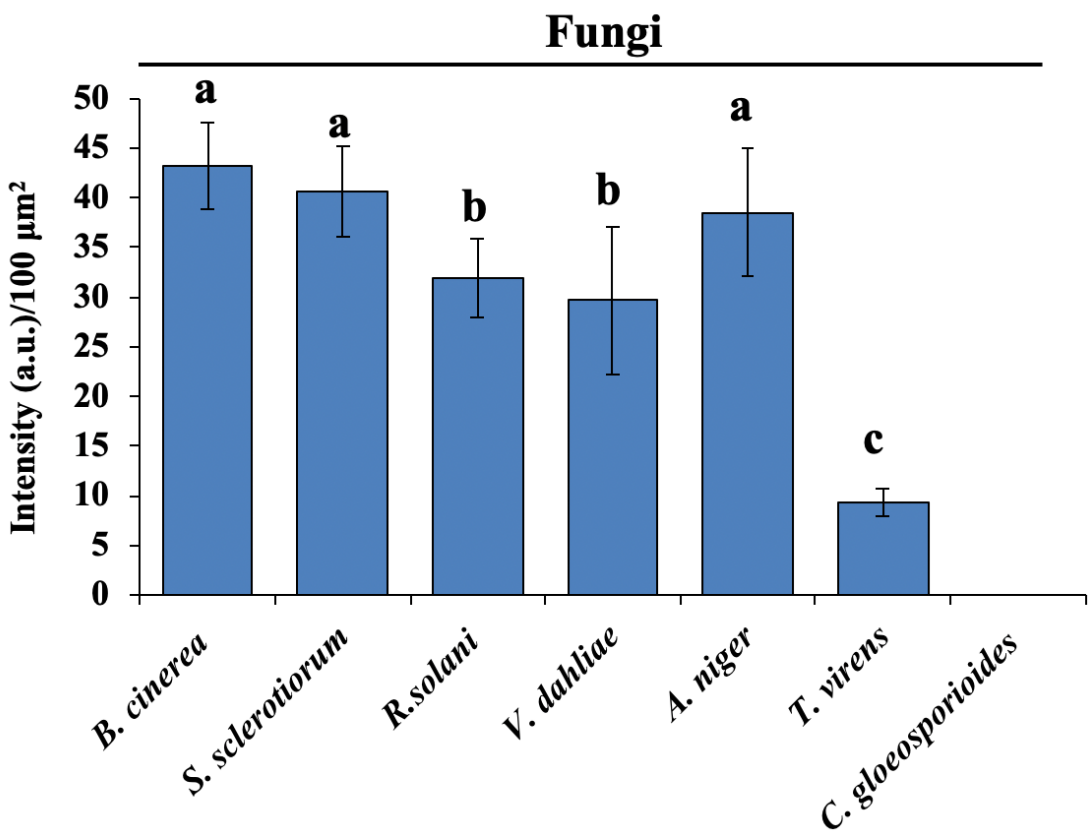

Figure S6. Accumulation of fluorescein signal intensities vary in different fungal cells.

The fluorescein signal intensities were quantified using ImageJ software, which could compare the fluorescence intensity of the same area between different cells. The letters above the bars indicate significant difference at a value of $P < 0.05$ (Duncan's multiple range test). Similar results were obtained from three biological repeats.

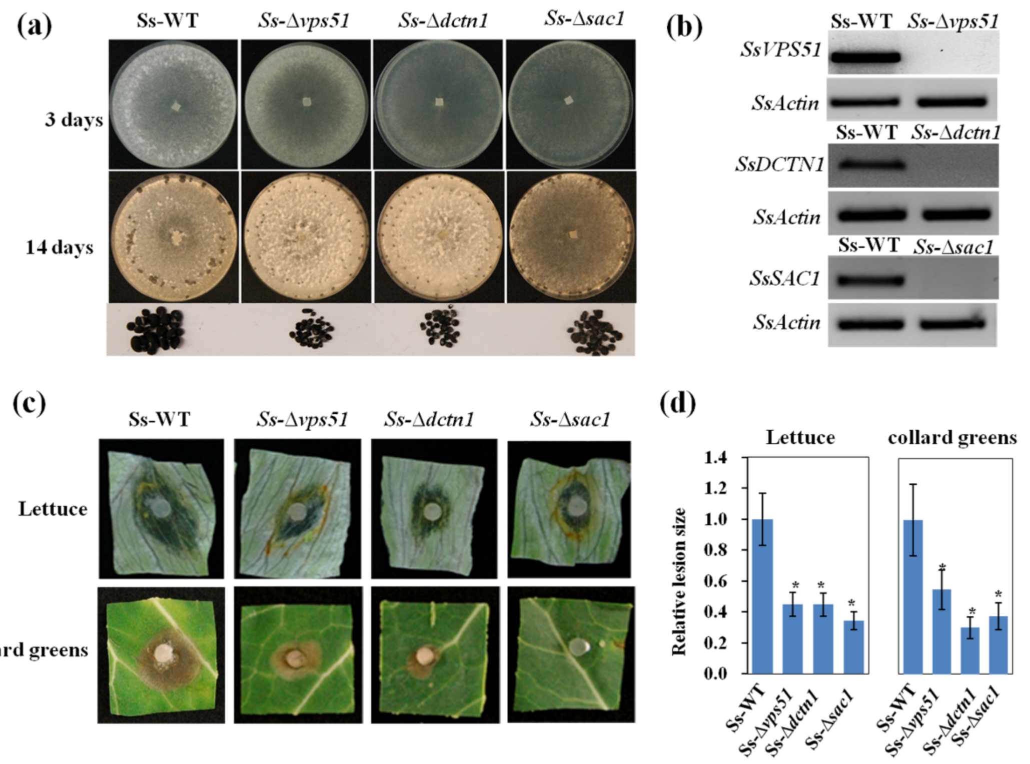

Figure S7. *SsVPS51*, *SsDCTN1*, and *SsSAC1* knockout mutants displayed significantly reduced virulence compared to the wild-type strain.

(a) Phenotypes of the *SsVPS51*, *SsDCTN1*, and *SsSAC1* knockout mutant strains grown on PDA solid medium at 25 °C. Photographs were taken after 3 and 14 days of fungal growth. **(b)** The expression levels of each gene in the knockout mutant strain were measured by RT-PCR. The *S. sclerotiorum Actin* gene was used as the internal expression control. Similar results were observed for the three biological replicates. **(c)** *Ss-Δvps51*, *Ss-Δdcnt1*, and *Ss-Δsac1* strains showed reduced virulence on lettuce and collard greens compared with Ss-WT. **(d)** The relative lesion sizes were measured at 3 dpi for lettuce and collard greens using ImageJ and error bars represent the SD of 10 plant samples. Asterisks (*) indicate statistically significant differences ($P < 0.05$, Student's t-test). Similar results were observed for the three biological replicates.

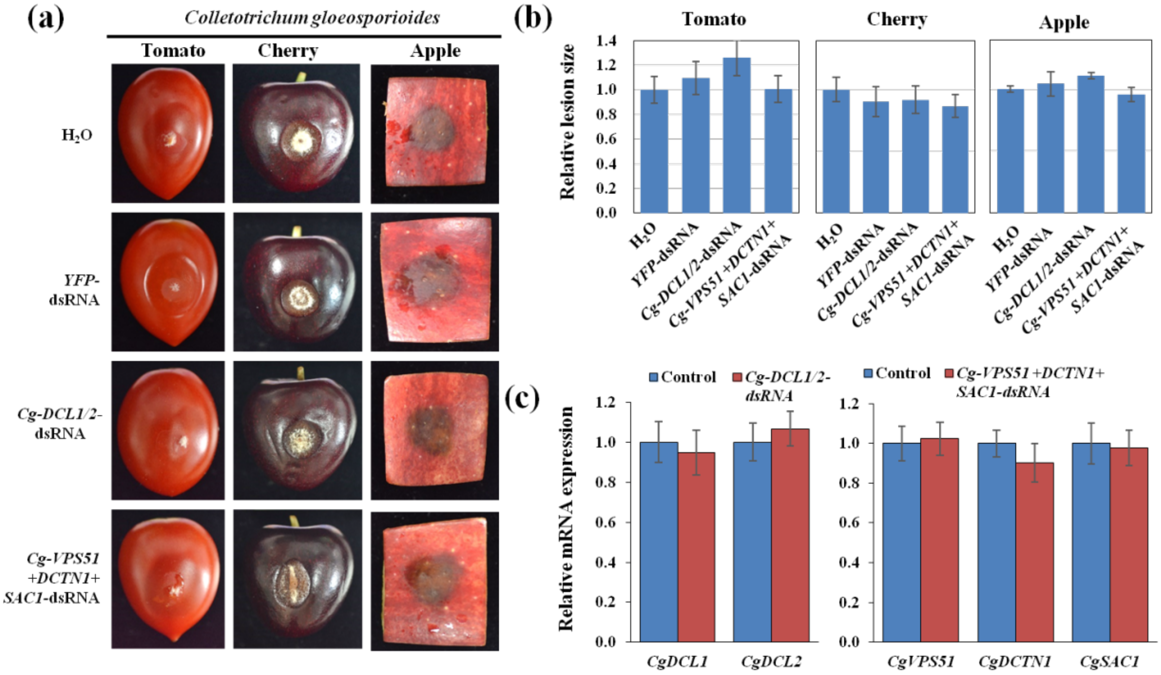

Figure S8. Topical application of pathogen gene-targeting dsRNAs had no effect on *C. gloeosporioides* infection and disease.

(a) Tomatoes, cherries, and apples were inoculated with *C. gloeosporioides* spores after treating with controls (water and *YFP*-dsRNA), *Cg-DCL1/2*-dsRNA or *Cg-VPS51+DCTN1+SAC1*-dsRNA (20 µl of 40 ng μl^{-1}). Pictures were taken at 5 dpi. **(b)** The relative lesion sizes were measured at 5 dpi using ImageJ software. Error bars indicate the SD of 10 samples. **(c)** The mRNA expression levels of *CgDCL1* and *CgDCL2* were detected in *Cg-DCL1/2*-dsRNA treated *C. gloeosporioides*, and *CgVPS51*, *CgDCTN1*, and *CgSAC1* were detected in *Cg-VPS51+DCTN1+SAC1*-dsRNA treated *C. gloeosporioides*. The expression levels of target genes were normalized to expression of *C. gloeosporioides Actin*. Error bars indicate the SD of three technical replicates. Similar results were observed from three biological replicates.

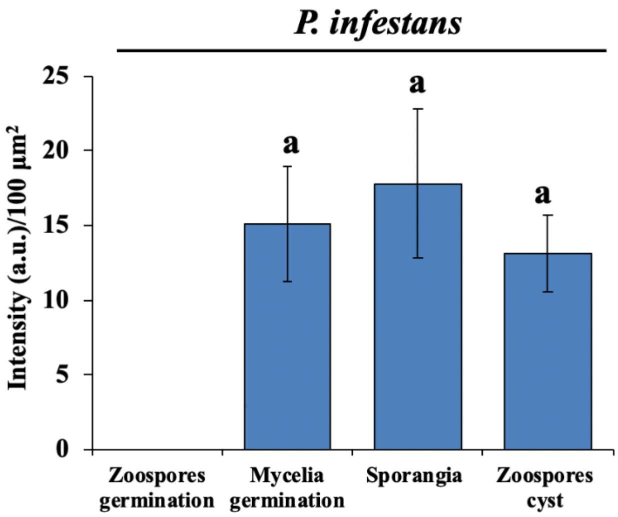

Figure S9. Accumulation of fluorescein signal intensities in different *P. infestans* cells.

The fluorescein signal intensities were quantified using ImageJ software, which could compare the fluorescence intensity of the same area between different cells. The letters above the bars indicate significant difference at a value of $P < 0.05$ (Duncan's multiple range test). Similar results were obtained from three biological repeats.

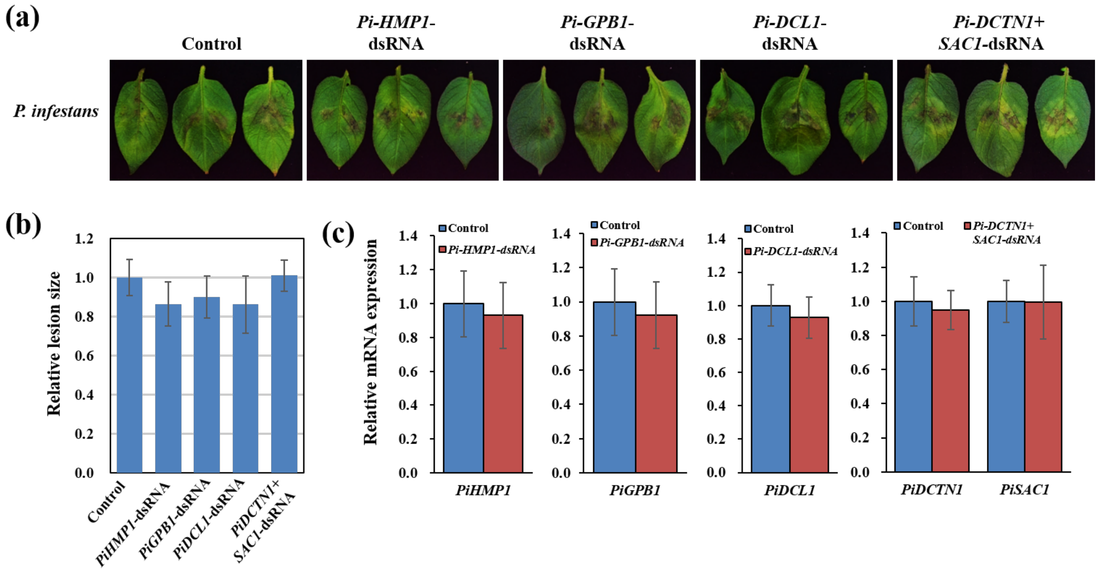

Figure S10. *YFP*-, *PiHMP1*-, *PiPGB1*-, *PiDCL1*-, and *Pi-DCTN1+SAC1*-dsRNAs did not inhibit *P. infestans* infection on potato leaves.

(a) Potato leaves were treated with *YFP*-, *PiHMP1*-, *PiPGB1*-, *PiDCL1*-, and *Pi-DCTN1+SAC1*-dsRNAs (40 ng μl^{-1}) and then inoculated with *P. infestans*, imaged at 3 dpi. **(b)** Relative lesion sizes were measured at 3 dpi using ImageJ software. Error bars indicate the SD of 10 samples. Similar results were observed in the three biological replicates. **(c)** The mRNA expression levels of *PiHMP1*, *PiPGB1*, *PiDCL1*, *PiDCTN1*, and *PiSAC1* were detected in corresponding dsRNA treated *P. infestans* by qRT-PCR analysis. The expression levels of target genes were normalized to expression of *P. infestans Tubulin*. Similar results were observed from three biological replicates.

Table S1. Primers used in the study

Primer Name	Primer Sequence (5′-3′)	Primer Description
YFP-dsRNA-T7-F	TAATACGACTCACTATAGGGAGA ATGGTGAGCAAGGGCGAGGA	Template DNA for *in vitro* *YFP*-dsRNA synthesis
YFP-dsRNA-T7-R	TAATACGACTCACTATAGGGAGA TTACTTGTACAGCTCGTCCATGC	
SsDCL1-dsRNA-T7-F	TAATACGACTCACTATAGGGAGA CATTTCGGAGGCGGTCG	Template DNA for *in vitro* *Ss-DCL1/2* synthesis
SsDCL1-dsRNA-T7-R	GATTATGTTCTGCCTCTTGCCAT GCCTTGGACATATTGAACAG	
SsDCL2-dsRNA-T7-F	CTGTTCAATATGTCCAAGGCATG GCAAGAGGCAGAACATAATC	
SsDCL2-dsRNA-T7-R	TAATACGACTCACTATAGGGAGA CGTAATATCATGTCCTCGG	
SsVPS51- dsRNA-T7-F	TAATACGACTCACTATAGGGAGA GCATTGGCTGCGCTGAAGAC	Template DNA for *In vitro* *Ss-VPS51+DCTN1+SAC1* synthesis
SsVPS51- dsRNA-T7-R	CCGTCAATCTCTGGCATTGACCT TGAAGAGTCTTTGTGAATC	
SsDCTN1-dsRNA-T7-F	GATTCACAAAGACTCTTCAAGGT CAATGCCAGAGATTGACGG	
SsDCTN1-dsRNA-T7-R	CCTGCTTGGAAATGTAAGCGTTT GGGTGATAGTTTGAGTC	
SsSAC1-dsRNA-T7-F	GACTCAAACTATCACCCAAACGC TTACATTTCCAAGCAGG	
SsSAC1-dsRNA-T7-R	TAATACGACTCACTATAGGGAGA CTGATAATGGCGATGCTATC	
SsVPS51-KO-5-F	GGGGTACCGGACGAATGGGAGT TATG	Construct *SsVPS5*1 ko mutant
SsVPS51-KO-5-R	CCCTCGAGCTCTTCGCCAACAAC CG	
SsVPS51-KO-3-F	AACTGCAGGTACATCTCGATTCC CAAAT	
SsVPS51-KO-3-R	CGGGATCCCTGTTAAGATATCTT AAATTTC	
SsDCTN1-KO-5-F	GGGGTACCCGTACCTTCCATTTT CCTC	Construct *SsDCTN1* ko mutant
SsDCTN1-KO-5-R	CCCTCGAGGTGTATACATGTTGC CTGC	
SsDCTN1-KO-3-F	AACTGCAGGTGTCAGCAATTCTA C	
SsDCTN1-KO-3-R	CGGGATCCCGGTTAACCAACATA GCC	
SsSAC1-KO-5-F	GGGGTACCCGTGCTCTTCTTGGC CTC	Construct *SsSAC1* ko mutant
SsSAC1-KO-5-R	CCCTCGAGCGGATGATGGTGAA GAATTTATTG	
SsSAC1-KO-3-F	CCCAAGCTTGGCAGCATGCTCTC AGC	
SsSAC1-KO-3-R	GGAATTCGTCGGACCAGCATCTT ATG	

SsVPS51-GT-5-F	CTTAGAGACTTGAGGAAGTC	*Ssvps51* ko mutant genotype identify
SsVPS51-GT-3-R	CTACTTCTATTGCTATTC	
SsDCTN1-GT-5-F	GATGAAGGAGTTCCCTTC	*Ssdctn1* ko mutant genotype identify
SsDCTN1-GT-3-R	CAGTACTAGATAATTCTAC	
SsSAC1-GT-5-F	CTTGGTGCCCTAGCATATC	*Sssac1* ko mutant genotype identify
SsSAC1-GT-3-R	CAACGAAGATCACGTATAAAG	
Ss-actin-qRT-F	GAGCTGTTTTCCCTTCCATTGTC	qRT-PCR Gene Expression
Ss-actin-qRT-R	GACGACACCGTGCTCGATTGG	
SsVPS51-qRT-F	CTGGTACAGGCTCTTTGC	qRT-PCR *SsVPS51* expression
SsVPS51-qRT-R	CACCTGATGTCTCCTGGTTCG	
SsDCTN1-qRT-F	GGATATGCCACCAGCAGGC	qRT-PCR *SsDCTN1* expression
SsDCTN1-qRT-R	CACACTGCCGACAAATCTG	
SsSAC1-qRT-F	GCTCGGGACACATTGAGAAG	qRT-PCR *SsSAC1* expression
SsSAC1-qRT-R	GCCACAGGTTCATCATTCTACC	
BcDCL1-dsRNA-T7-F	TAATACGACTCACTATAGGGAGATGCGGAAGAACTTGAAGGTTTGCTACA	Template DNA for *In vitro Bc-DCL1/2* synthesis
BcDCL1-dsRNA-T7-R	GCAGCAAATGGCATCCGTCCAGATCTGGTCAACACACCAAG	
BcDCL2-dsRNA-T7-F	CTTGGTGTGTTGACCAGATCTGGACGGATGCCATTTGCTGC	
BcDCL2-dsRNA-T7-R	TAATACGACTCACTATAGGGAGAACTCTTGAGTACTTTCGCCAGCTCAC	
BcVPS51-dsRNA-T7-F	TAATACGACTCACTATAGGGAGATTCGTTCCAGGAGTTACACG	Template DNA for *In vitro Bc-VPS51+DCTN1+SAC1* synthesis
BcVPS51-dsRNA-T7-R	CTTTCGACGAGAGCACGAATGATGAGACAAGTGAGAGTCCA	
BcDCTN1-dsRNA-T7-F	TGGACTCTCACTTGTCTCATCATTCGTGCTCTCGTCGAAAG	
BcDCTN1-dsRNA-T7-R	CACTGCACTTTGAACAACGTCCAGCTTACAACTGTGCTCT	
BcSAC1-dsRNA-T7-F	AGAGCACAGTTGTAAGCTGGACGTTGTTCAAAGTGCAGTG	
BcSAC1-dsRNA-T7-R	TAATACGACTCACTATAGGGAGACCTTCAATGCTGCTGTAGAAG	
Bc-actin-qRT-F	TGCTCCAGAAGCTTTGTTCCAA	qRT-PCR Gene Expression
Bc-actin-qRT-R	TCGGAGATACCTGGGTACATAG	
BcDCL1-qRT-F	ACAATCCTATCTTTCGGAAGC	qRT-PCR *BcDCL1* Expression
BcDCL1-qRT-R	AGACTCTTCTTCTTGAAGACAG	
BcDCL2-qRT-F	GATTGTGCAAAGTCTCAACA	qRT-PCR *BcDCL2* Expression
BcDCL2-qRT-R	ATTGGGTTTGACTATATGTCTTA	
BcVPS51-qRT-F	GTTAGGCAAAGTAGGAGAAGG	qRT-PCR *BcVPS51* Expression
BcVPS51-qRT-R	CATCCATTCGCCCCAATACC	
BcDCTN1-qRT-F	GCTTCCCTTACCTCGAATTC	qRT-PCR *BcDCTN1* Expression
BcDCTN1-qRT-R	CAACCTCTAGTTCTTCCGGTC	
BcSAC1-qRT-F	CGAAAAGTTTCGAGGCATTTG	qRT-PCR *BcSAC1* Expression
BcSAC1-qRT-R	GAACTTAATGGGGTCAAAGCC	
CgDCL1-dsRNA-T7-F	TAATACGACTCACTATAGGGAGAGTCAGATGTCTATCCCGTTTCCTT	Template DNA for *in vitro*

	G	*Cg-DCL1/2* synthesis
CgDCL1-dsRNA-T7-R	CTCGATGGGAAGCTGATCACCGA CTTCAGAAAGCTTTGCCG	
CgDCL2-dsRNA-T7-F	CGGCAAAGCTTTCTGAAGTCGGT GATCAGCTTCCCATCGAG	
CgDCL2-dsRNA-T7-R	TAATACGACTCACTATAGGGAGA GAAGCAGGTGAGGTCGTGTGC	
CgVPS51-dsRNA-T7-F	TAATACGACTCACTATAGGGAGA GTCTGGTCGTGCTGAGGAAAC	Template DNA for *in vitro* *Cg-VPS51+DCTN1+SAC1* synthesis
CgVPS51-dsRNA-T7-R	GCGCTTGTGAAGCTGGTCGCGCA GCTCCTCTGACGCGGAG	
CgDCTN1-dsRNA-T7-F	CTCCGCGTCAGAGGAGCTGCGCG ACCAGCTTCACAAGCGC	
CgDCTN1-dsRNA-T7-R	GTCCAACTTGTCGCGGAGATCGG CGTAAGACTCGCTCAC	
CgSAC1-dsRNA-T7-F	GTGAGCGAGTCTTACGCCGATCT CCGCGACAAGTTGGAC	
CgSAC1-dsRNA-T7-R	TAATACGACTCACTATAGGGAGA GTCAACCTGAGCAGCAAGATC	
Cg-Actin-qRT-F	CTCAGAGGCCTGCTGAAGATC	qRT-PCR Gene Expression
Cg-Actin-qRT-R	GCTTGGATTGATGTGTACAGTGC	
CgDCL1-qRT-F	TACTGACACGACGATTT	qRT-PCR *CgDCL1* Expression
CgDCL1-qRT-R	TTGAAGTTCCTCCCAC	
CgDCL2-qRT-F	CCGAACCTCAAATCC	qRT-PCR *CgDCL2* Expression
CgDCL2-qRT-R	CCGCCTCTTCAATCT	
CgVPS51-qRT-F	GACACTCACGTCCTCCG	qRT-PCR *CgVPS51* Expression
CgVPS51-qRT-R	TTGTCCTTCCGCATCT	
CgDCTN1-qRT-F	GAAGGCTATCATCCACTC	qRT-PCR *CgDCTN1* Expression
CgDCTN1-qRT-R	CTACGCAGCATCTCCA	
CgSAC1-qRT-F	GAGCCGACCCTGTTA	qRT-PCR *CgSAC1* Expression
CgSAC1-qRT-R	ACTAGAGGCCGCATT	
RsPG-dsRNA-T7-F	TAATACGACTCACTATAGGGAGA TGACAGCAAGTACAGGT	Template DNA for *in vitro* *Rs-PG* synthesis
RsPG-dsRNA-T7-R	TAATACGACTCACTATAGGGAGA TCACACTGGTGCCCGAA	
RsDCTN1-dsRNA-T7-F	TAATACGACTCACTATAGGGAGA GTTGCCTCGCACTAC	Template DNA for *in vitro* *Rs-DCTN1+SAC1* synthesis
RsDCTN1-dsRNA-T7-R	CCCACTTCAAGTCGGTCGCAGTC ACACCAA	
RsSAC1-dsRNA-T7-F	TTGGTGTGACTGCGACCGACTTG AAGTGGG	
RsSAC1-dsRNA-T7-R	TAATACGACTCACTATAGGGAGA CCGCCTGAGTCTGTA	
Rs-Actin-qRT-F	CCAACCGAGAAAAGATGACGC	qRT-PCR Gene Expression
Rs-Actin-qRT-R	CGTAAATTGGAACCGTATGCG	
Os18S-rRNA-qRT-F	ATGATAACTCGACGGATCGC	qRT-PCR Gene Expression
Os18S-rRNA-qRT-R	CTTGGATGTGGTAGCCGTTT	
RsPG-qRT-F	CGCCGAAGTGGATGT	qRT-PCR *RsPG* Expression
RsPG-qRT-R	TTGGACGAATTTCAGATAG	
RsDCTN1-qRT-F	TAGGGAGGGTGCTGT	qRT-PCR *RsDCTN1* Expression
RsDCTN1-qRT-R	AAATCTGTCTCCTGCTTC	

RsSAC1-qRT-F	CAATGATGCCACTCC	qRT-PCR *RsSAC1* Expression
RsSAC1-qRT-R	ATCTCGGCTCGTTAG	
AnpgxB-dsRNA-T7-F	TAATACGACTCACTATAGGGAGACGACGATGTGTCCAGTGACTTC	Template DNA for *in vitro AnpgxB* synthesis
AnpgxB-dsRNA-T7-R	TAATACGACTCACTATAGGGAGAGCAGGACCACCGAAATCAGTATC	
AnVPS51-dsRNA-T7-F	TAATACGACTCACTATAGGGAGAGCCATGATGAAGATGCCGC	Template DNA for *in vitro An-VPS51+DCTN1+SAC1* synthesis
AnVPS51-dsRNA-T7-R	GACCGGACGAGCGCTTGGTGACGGACTTCGCCGAAGGGACGC	
AnDCTN1-dsRNA-T7-F	GCGTCCCTTCGGCGAAGTCCGTCACCAAGCGCTCGTCCGGTC	
AnDCTN1-dsRNA-T7-R	CTCCAGATGCTTCACTAGGCGGTCAAGGTGAGCTTGGACGGC	
AnSAC1-dsRNA-T7-F	GCCGTCCAAGCTCACCTTGACCGCCTAGTGAAGCATCTGGAG	
AnSAC1-dsRNA-T7-R	TAATACGACTCACTATAGGGAGACGATGTTGATGCCCTCGC	
An-Actin-qRT-F	ACGCTTGGACTGTGCCTC	qRT-PCR Gene Expression
An-Actin-qRT-R	CAATGGTTCGGGTATGTGC	
AnpgxB-qRT-F	TTCGGCGAGAAGCAGTCA	qRT-PCR *AnpgxB* Expression
AnpgxB-qRT-R	CACCGATAATGCAACCCG	
AnVPS51-qRT-F	CACAAACTGGCTGGAC	qRT-PCR *AnVPS51* Expression
AnVPS51-qRT-R	GCCTTGACCGAATACA	
AnDCTN1-qRT-F	GACGAGCAAGCACATAC	qRT-PCR *AnDCTN1* Expression
AnDCTN1-qRT-R	GCAGCGATCTCCAAC	
AnSAC1-qRT-F	CCCATTTATGCCGTGAC	qRT-PCR *AnSAC1* Expression
AnSAC1-qRT-R	GTGCCAACTTCAGTGTCG	
VdDCL1-dsRNA-T7-F	TAATACGACTCACTATAGGGAGATCATGACCACAGTCATGAG	Template DNA for *in vitro Vd-DCL1/2* synthesis
VdDCL1-dsRNA-T7-R	GTCGCCTGTGGCAGTAGATCGTTGACCTTCTGTGTATGG	
VdDCL2-dsRNA-T7-F	CCATACACAGAAGGTCAACGATCTACTGCCACAGGCGAC	
VdDCL2-dsRNA-T7-R	TAATACGACTCACTATAGGGAGACCAGTCGAACCTCGACAATG	
VdDCTN1-dsRNA-T7-F	TAATACGACTCACTATAGGGAGACTCGTCACGCCTGGCGCC	Template DNA for *in vitro Vd-DCTN1+SAC1* synthesis
VdDCTN1-dsRNA-T7-R	CTTGGTCCTCATGGCATCCAGCAGGCTGGCGGCGCGAGGCGTC	
VdSAC1-dsRNA-T7-F	GACGCCTCGCGCCGCCAGCCTGCTGGATGCCATGAGGACCAAG	
VdSAC1-dsRNA-T7-R	TAATACGACTCACTATAGGGAGAGCGGATTGCTGGTCTACTTGCATG	
At-actin2- qRT-F	ACCTTGCTGGACGTGACCTTACTGAT	qRT-PCR Gene Expression
At-actin2- qRT-R	GTTGTCTCGTGGATTCCAGCAGCTT	
Vd-actin-qRT-F	TAACTCTCCCGCTATGTATGTCGC	qRT-PCR Gene Expression
Vd-actin-qRT-R	GAGAGAAACCCTCGTAGATTGGC	

VdDCL1-qRT-F	GTCGCAGACCTTCCATACTT	qRT-PCR *VdDCL1* Expression
VdDCL1-qRT-R	AGATCTGTCCGAATACCTCTGAG	
VdDCL2-qRT-F	GACTCTCCAGGCTTTCAAGA	qRT-PCR *VdDCL2* Expression
VdDCL2-qRT-R	TCTGAGAAATTGAGGTCACGAA	
VdDCTN1-qRT-F	CGTTGGCTCTGTTTCCT	qRT-PCR *VdDCTN1* Expression
VdDCTN1-qRT-R	GGTTCTCCATCAGCGTCT	
VdSAC1-qRT-F	TTCTGGAACCGAAATAC	qRT-PCR *VdSAC1* Expression
VdSAC1-qRT-R	CGGAGATGTTGGAGC	
PiDCL1-dsRNA -T7-F	TAATACGACTCACTATAGGGAGA GAAGTTCGTGGAGCTAGAGC	Template DNA for in vitro *PiDCL1* synthesis
PiDCL1-dsRNA -T7-R	TAATACGACTCACTATAGGGAGA GCCTTCTTTACCAGCGCTATC	
PiHMP1-dsRNA -T7-F	TAATACGACTCACTATAGGGAGA GGCTCCGGCCGACATCCCG	Template DNA for in vitro *PiHMP1* synthesis
PiHMP1-dsRNA -T7-R	TAATACGACTCACTATAGGGAGA GTGTCTTTAGTAAAGTCACCG	
PiGPB1-dsRNA -T7-F	TAATACGACTCACTATAGGGAGA ATGGGCGACGCTGCGGAGC	Template DNA for in vitro *PiGPB1* synthesis
PiGPB1-dsRNA -T7-R	TAATACGACTCACTATAGGGAGA CGACATTTGGGTGGGGCCAG	
PiDCTN1-dsRNA-T7-F	TAATACGACTCACTATAGGGAGA CAGCTTCAGCAGCAACAACAG	Template DNA for *in vitro Pi-DCTN1+SAC1* synthesis
PiDCTN1-dsRNA-T7-R	GTCACGACAGTATCAGAACGCG AAGAAGTAGGTTCCGCTGC	
PiSAC1-dsRNA-T7-F	GCAGCGGAACCTACTTCTTCGCG TTCTGATACTGTCGTGAC	
PiSAC1-dsRNA-T7-R	TAATACGACTCACTATAGGGAGA CGTGCACGTGCTGATTATCC	
Pi-Tubulin-qRT-F	GAATACGATCCCACGAA	qRT-PCR Gene Expression
Pi-Tubulin-qRT-R	AACGAGCCCAGTCCA	
PiDCL1-qRT-F	GCGTGTCGTGTTTGA	qRT-PCR *PiDCL1* Expression
PiDCL1-qRT-R	TTTCAGCACGTCAATT	
PiGPB1-qRT-F	ATTTCTTCCCGAGTGG	qRT-PCR *PiGPB1* Expression
PiGPB1-qRT-R	TTGTAGTCGTCGTAGCC	
PiHMP1-qRT-F	GTTTCCGCCGTCGTT	qRT-PCR *PiHMP1* Expression
PiHMP1-qRT-R	TCCGTGTCCGTAGTCG	
PiDCTN1-qRT-F	CTCTGTTTCCCAAGCG	qRT-PCR *PiDCTN1* Expression
PiDCTN1-qRT-R	ATCGCCCACATCCAA	
PiSAC1-qRT-F	CAGTAGTAGTGGGAGCG	qRT-PCR *PiSAC1* Expression
PiSAC1-qRT-R	GAGCGACACTTTGAGC	

Table S2. The sequences of dsRNAs in this study.

dsRNA	dsRNA sequence
YFP-dsRNA	TAATACGACTCACTATAGGGAGAATGGTGAGCAAGGGCGAGGAGCTGT<br>TCACCGGGGTGGTGCCCATCCTGGTCGAGCTGGACGGCGACGTAAACGG<br>CCACAAGTTCAGCGTGTCCGGCGAGGGCGAGGGCGATGCCACCTACGG<br>CAAGCTGACCCTGAAGTTCATCTGCACCACCGGCAAGCTGCCCGTGCCC<br>TGGCCCACCCTCGTGACCACCTTCGGCTACGGCGTGCAGTGCTTCGCCC<br>GCTACCCCGACCACATGCGCCAGCACGACTTCTTCAAGTCCGCCATGCC<br>CGAAGGCTACGTCCAGGAGCGCACCATCTTCTTCAAGGACGACGGCAAC<br>TACAAGACCCGCGCCGAGGTGAAGTTCGAGGGCGACACCCTGGTGAAC<br>CGCATCGAGCTGAAGGGCATCGACTTCAAGGAGGACGGCAACATCCTG<br>GGGCACAAGCTGGAGTACAACTACAACAGCCACAACGTCTATATCATG<br>GCCGACAAGCAGAAGAACGGCATCAAGGTGAACTTCAAGATCCGCCAC<br>AACATCGAGGACGGCAGCGTGCAGCTCGCCGACCACTACCAGCAGAAC<br>ACCCCCATCGGCGACGGCCCCGTGCTGCTGCCCGACAACCACTACCTGA<br>GCTACCAGTCCGCCCTGAGCAAAGACCCCAACGAGAAGCGCGATCACA<br>TGGTCCTGCTGGAGTTCGTGACCGCCGCCGGGATCACTCTCGGCATGGA<br>CGAGCTGTACAAGTAAAGAGGGATATCACTCAGCATAAT
Ss-DCL1/2-dsRNA	TAATACGACTCACTATAGGGAGACATTTCGGAGGCGGTCGAAACTCGCC<br>GGTTTCATGCGTGCCTCTCACTTCTTCAGTGAAGCTCGAAGGAACCACG<br>CTTGACCAAGTTAATATGTTCACCCTATGTCTATTTCAGGATGTGTTCAG<br>CAAAGCTTACAAATCGAATCCTGATAGTATGCCGTACTTTTTGGTTCCTA<br>TCAATTGTCCAAATCACACTGGGGACTGGAAAGATTATGAGCCCATGTC<br>AATAATTGATTGGGAAACTGTTCAATATGTCCAAGGCATGGCAAGAGGC<br>AGAACATAATCCTGGAATTGTTCGTACATCTATTATGAGCCTCGATGATT<br>TAAAGATTGAGCTCTGCCTTCCGATCGAGCCACCACCAATTCCGCCCTT<br>GACACTTCACTGGAATTCCAGCACAGAGCTGTCTGTCGAGTTTACAAAC<br>GATGCCGAAATAGGAACCACCGAGGACATGATATTACGAGAGGGATAT<br>CACTCAGCATAAT
Ss-<br>*VPS51+DCTN1+SAC1*<br>-dsRNA	TAATACGACTCACTATAGGGAGAGCATTGGCTGCGCTGAAGACGAAAC<br>CTTTATTGAAAGATGAAAGTATTCGAGATTTGGAGGGATTGAGATTAGA<br>TGTATGTGAGCGGTGGTTTGGTGATGAAATTCTTTACTTTACACCTTATG<br>TTCGACACGATGATTTGGAAGGATCATTAGCCGTTGAGACATTAAGAGG<br>TTGGGCGAAGAAAGCATCAGAGGTACTACTGGAAGGATTCACAAAGAC<br>TCTTCAAGGTCAATGCCAGAGATTGACGGTTACTCTTCAGCAGCGGAGG<br>ATTTCTGTGAATTGTGCGAGGCATCAGGTCATGATGTTCTACATTGCCCC<br>ATGTTCGGTCCTAATGGTAATAGTGGCAACTCTAGAGAGGAGTCTCCTA<br>AAGAGCAACGAACAGGAAAAGACGTTGTCATGGAAGGACTCAAACTAT<br>CACCCAAACGCTTACATTTCCAAGCAGGAAATCAATGGCCGCTTGACTG<br>AAATAATCATTAACAATACCGCTGTAAAATCTAGAGATAGAAAGTCCCA<br>TGTCTGTCAAAGCACCCTTGTAATCACGCTTTCTAGTACGAGTGAAGTCT<br>CCCTTCAGGGCTGCTGTAGAGGCATATTGCTTAGAGATAGCATCGCCAT<br>TATCAGAGAGGGATATCACTCAGCATAAT
Bc-DCL1/2-dsRNA<br>(Wang et al., 2016)	TAATACGACTCACTATAGGGAGATGCGGAAGAACTTGAAGGTTTGCTAC<br>ACAGTCAAATATGTACTGCAGAAGATCCCAGCTTGCTGCAGTACTCAAT<br>CAAAGGTAAACCTGAGACTCTTGCCTACTATGATCCCTTGGGCCCGAAA<br>TTCAATACTCCTCTTTATCTTCAAATGCTCCCGCTTCTAAAAGACAATCC<br>TATCTTTCGGAAGCCATTTGTATTTGGGACAGAAGCCAGTAGAACTCTA<br>GGATCTTGGTGTGTTGACCAGATCTGGACGGATGCCATTTGCTGCACGC<br>CAAAAATACATCGAGCAGATCTTCGCCTTCGAGTAAAGCTACCACTTCT<br>ATCTATTATCTACTATACCCCAGAGTCAAATATCATCGTGACGAAAACT<br>GTGGCGAGCCTGAGAAAGATTGTGCAAAGTCTCAACATTTTCGAAGACC<br>CCTACGTTTTGACACTAAAAAGGAGTGATAGCGAAAAAAGTCAACGTG<br>AGCTGGCGAAAGTACTCAAGAGTAGAGGGATATCACTCAGCATAAT
Bc-	TAATACGACTCACTATAGGGAGATTCGTTCCAGGAGTTACACGCAAAGA

VPS51+DCTN1+SAC1<br>-dsRNA	TCACTTCACTGGTGGACCAGCAAAAAGATAGTAAACATATCGGGAAAA<br>TATCGATATATATTCTCCGAATTCTACGAGATATCAGAGCAGAATTACC<br>TAGTAACCCTGCACTACAAAAGTTTGGACTCTCACTTGTCTCATCATTCG<br>TGCTCTCGTCGAAAGCAAAGACAATAAAGAAGAACAAATGGAGCGCGA<br>GTTGGAGGGATTGCGAAGAGGAAGTGTTAGCAATCCTACTACGCATCGT<br>ACTAGTGCCATGAGCAGCGGAACTGTGACTCAGGATAGGAATTCTCTCC<br>AAGACAATAAGAGCACAGTTGTAAGCTGGACGTTGTTCAAAGTGCAGT<br>GGCAAAGCGAGCACTTGAAATGCAGTTAAAGAATGAGGGACTAGATGT<br>CACTCTACAAATTGATCAAACTCAACAATGGTTCAATACTTTGTGGGCC<br>GACAATGGTGACGCCATTTCTAAGCAATACGCTTCTACAGCAGCATTGA<br>AGGAGAGGGATATCACTCAGCATAAT
Cg-DCL1/2-dsRNA	TAATACGACTCACTATAGGGAGAGTCAGATGTCTATCCCGTTTCCTTGCC<br>CACTCCTATCCGGGTGTCTGCAGAAGACTCCGAAGCCCTGAAAGAGTTT<br>ACCTTACGCATTTTCAAAGACGTCTTCAGCAAGCAGTTCGAGGCCCCAA<br>TTACTGACGTGCCGTACTTTCTTGCCCCTGCCACATTCTATCACTCGGCA<br>AAGCTTTCTGAAGTCGGTGATCAGCTTCCCATCGAGGAGCCTGAGAACG<br>GAGACCATACTGCTGTACTTCTCGCCTTGCCTTACGGCCATCGCTGGTTG<br>TCGAGTCCGCTTCAGCAGGTAATCAGGTTTAGCGCAGTTGGTGCCGAAC<br>TTTCTCTGGATGGTATTGGAACGAAGCATTTTGTGCCAGGGGAGATGTC<br>TGCACACGACCTCACCTGCTTCAGAGGGATATCACTCAGCATAAT
Vd-DCL1/2-dsRNA<br>(Wang et al., 2016)	TAATACGACTCACTATAGGGAGATCATGACCACAGTCATGAGTTCTCAC<br>CGAATGAAGACCCAGGGTCACTGATCGACTGGAGCCATCTGCTGTCGAC<br>CAAAGAGGTTGAGTACTTGCCTTGGGATGAAGATCACAGTCCCAGCTTC<br>TATCAAAGCAAGTTTGTGATTGATCCATACACAGAAGGTCAACGATCTA<br>CTGCCACAGGCGACAAACAAGAAGCCATATGCTAGGGTTTATCCGGCAC<br>CGTGGGCGAAAGTCGACACGATTCCATTAGATCATGCTTACTTTGGGGC<br>GTTGATCCCTTTCATTTCACACATTGTCGAGGTTCGACTGGAGAGGGAT<br>ATCACTCAGCATAAT
PiHMP1-dsRNA	TAATACGACTCACTATAGGGAGAGGCTCCGGCCGACATCCCGTCTTCAC<br>AAGACGAGAATCTCAAGAAACAGGAAGAGCGTGGCTTCTTTGACTGGTT<br>TGGCAATGGCGACGACACGCCTGCCCCGGCTGCTGACGACAACTCCGGT<br>AAATATGCGACTACGACACCGATCCCTGGCACGGCTGCGCGGCCGTCTA<br>CGAGTAGTCTGATGTCAAAGTATGGTAGTATGCTCGGTGACTTTACTAA<br>AGACACGAGAGGGATATCACTCAGCATAAT
PiPGB1-dsRNA	TAATACGACTCACTATAGGGAGAATGGGCGACGCTGCGGAGCTCAAGA<br>AGAAATGCGAGAGCCTCAAGGAGACGATTGAGAAGACGCGTGAAGCCA<br>AGAGTGATGGCGGCTTCCAGAGCGCCAATGCCAGCTCCGGCGCCAAGG<br>CGATCCTGGCCCCACCCAAATGTCGAGAGGGATATCACTCAGCATAAT
PiDCL1-dsRNA	TAATACGACTCACTATAGGGAGAGAAGTTCGTGGAGCTAGAGCTGCAG<br>GCCAATTTACAGGCGTGTATTGTGGACGACCCAGACAACGTGAACAACA<br>GGAAGAAGCGCAAGCAGCAAGAAGACGATCCTGATATCGACGATTCGA<br>TGATGGGAGAGGGCATTGATAGCGACTTCACGGAAGATGGAGCGGTGG<br>GTGAGGAAGAGGGCACGGACGAGATGATGGAGTGCAGCAATGCAGAA<br>GATAGCGCTGGTAAAGAAGGCAGAGGGATATCACTCAGCATAAT

"_" representative T7 promoter sequence, color representative different genes sequence fragments that used for dsRNA templates.

Reference

Wang, M., Weiberg, A., Lin, F.M., Thomma, B.P., Huang, H.D. and Jin, H. (2016) Bidirectional cross-kingdom RNAi and fungal uptake of external RNAs confer plant protection. *Nature plants* **2**, 16151.
